# The SHARE-HRS 4S^2^ Model of Surge Capacity in Humanitarian Health Care Response Settings: A Revised Model Informed by Lived Experiences

**DOI:** 10.1017/S1049023X25101210

**Published:** 2025-06

**Authors:** Zachary B. Horn, Andrea P. Marshall, Jamie Ranse

**Affiliations:** 1. School of Nursing and Midwifery, Griffith University, Gold Coast, Queensland, Australia; 2. School of Medicine and Dentistry, Griffith University, Gold Coast, Queensland, Australia; 3. Nursing and Midwifery Education and Research Unit, Gold Coast Health, Gold Coast, Queensland, Australia; 4. Department of Emergency Medicine, Gold Coast Health, Gold Coast, Queensland, Australia

**Keywords:** content analysis, disaster, disaster medicine, humanitarian, surge capacity

## Abstract

**Introduction:**

Conceptualizations of surge capacity are gaining traction in disaster preparedness and response, particularly in the context of critical and acute care during the pandemic as well as other disaster contexts. In most applications, the surge capacity domains describe the four types of assets required to ensure that surges in demand are addressed. Despite increasing interest and conceptual application, these constructs are yet to be considered or explored in relation to the profound resource scarcity and complex contexts of humanitarian health responses.

**Objectives:**

The aim of this research is to explore surge capacity domain constructs in the novel context of scarce health resource allocation in humanitarian health care response settings.

**Methods:**

This research was conducted according to an exploratory qualitative design. Clinicians and managers with relevant experiences were purposively recruited to include broad perspectives across humanitarian responses and clinical specialties. Interview transcripts were analyzed using a latent deductive pattern approach, using a deductive code book consisting of existing surge capacity domains to explore surge capacity constructs. Analysis of coded data for cross-cutting themes drove identification of new findings regarding surge capacity in the context of humanitarian health responses.

**Results:**

Seventeen participants completed semi-structured interviews. In addition to demonstrating the relevance of existing surge capacity domains (staff, stuff, space, and systems; 4Ss), four new themes emerged: (1) sponsorship; (2) suitability; (3) security; and (4) supply. These four themes informed the conceptualization of surge capacity dimensions which must be satisfied for an asset to render a positive impact with relevance to all four surge capacity domains (4S^2^ - cumulative 4S domains and the new dimensions).

**Conclusions:**

Although existing surge capacity domains have proven relevant to humanitarian health care response settings, this research produced a revised conceptualization of surge capacity constructs specific to this context. The identification of four surge capacity dimensions supported the conception and development of the Scarce Health Resource Allocation in Humanitarian Response Settings (SHARE-HRS) 4S^2^ model of surge capacity, thus offering a potential new tool to support humanitarian health response planning and evaluation.

## Introduction

Surge capacity has been defined as “the ability to obtain adequate staff, supplies and equipment, structures, and systems to provide sufficient care to meet immediate needs of an influx of patients following a large-scale incident or disaster.”^1^ Conceptually and operationally, surge capacity has become accepted as four essential categories, or domains, of resources referred to the “four Ss”: (1) staff; (2) stuff or supplies; (3) space of structures; and (4) systems (4S).^1–3^ The relationship between surge capacity and a need for additional health care, thus the surge itself, is conceptualized by Kelen & McCarthy as the product of resources across the “four Ss” divided by the product of event, influx, and resource demand.^3^

Surge capacity has become an attractive concept in disaster planning to guide preparedness for the continuation of health services following disasters and associated surges in demand. The COVID-19 pandemic serves as a prominent recent example for large-scale surges in demand and brought acute attention to surge capacity conceptualizations in emergency and intensive care services. Particular attention was paid to the extension of system capacity to respond to increased demand for critical care and invasive organ support, as demonstrated by several studies which utilized the 4Ss to structure recommendations or evaluations.^4–8^

Despite growing interest and application in health care settings and relative resource scarcity, the formal translation of surge capacity beyond hospital settings to humanitarian health response settings remains mostly unexplored. Humanitarian crises and complex humanitarian emergencies, by their nature, involve critical health resource shortages generated or compounded by violence, armed conflict, and political breakdown.^9^ Given the role of surge capacity in guiding the enhancement of health system preparedness and response, these constructs offer an attractive potential contribution to the preparedness and response capabilities of humanitarian health responses.

Through a systematic mapping review and evidence gap map (EGM), it was identified that surge capacity domains could be applied to existing accounts of scarce health resource allocation in humanitarian response settings while demonstrating a significant lack of intentional and quality research conducted in this area.^10^ Additionally, in the review, it was identified that existing conceptualizations of surge capacity are likely incomplete when considering humanitarian response settings, demonstrated by the emergence of “security” as a theme that cut across existing surge capacity domains.^10^ It was therefore identified that further evidence was required to address the specific function of security and that focused exploration of surge capacity constructs in humanitarian response settings is required.

The aim of this research was to explore clinicians’ lived experiences of participating in scarce health resource allocation in humanitarian response settings, and thus address the identified gap in the surge capacity literature.

## Methods

### Design

The overall project was in keeping with a cross-sectional exploratory qualitative design informed by pragmatist and phenomenological perspectives and utilizing three independent layers of data analysis. This study utilized participant narrative generated in the course of a larger research project, referred to as Scarce Health Resource Allocation in Humanitarian Response Settings (SHARE-HRS). The findings here arise exclusively from the described analytic approach driven to address the specified research aim; therefore, the contents of this report represent a unique and focused research output not otherwhere reported.

### Participant Sample

Participants were purposively identified and recruited to achieve maximum variation sampling. Participants were identified through academic publications, mainstream media outputs, and publicly available professional profiles (such as LinkedIn; Mountain View, California USA) as having participated in humanitarian responses. The primary requirement for recruitment was first-hand experience in scarce health resource allocation in humanitarian response settings. Characteristics such as gender, clinical role/profession, response organization, and response characteristics (location, nature of inciting, and potentiating hazards/events) were all considered during purposive sampling to maximize the spread of characteristics covered by the sample. Participant recruitment was terminated upon reaching adequate participant power and information power in that the sample and data were sufficient to support the emerging findings; therefore, recruitment termination was driven by more comprehensive considerations and reflections associated with the goal of data saturation alone.^11–14^

### Data Collection

Interviews were conducted with individual participants using a semi-structured interview guide (Supplementary File 1; available online only). Interviews were hosted remotely (via Microsoft Teams; Redmond, Washington USA) and were recorded to facilitate transcription through a combination of the built-in speech-to-text function and manual transcription.

### Data Analysis

Data were analyzed in accordance with deductive latent content analysis.^15–17^ The deductive codebook consisted of the existing surge capacity domains (staff, space, stuff, and systems; 4Ss) in keeping with already presented definitions (Table 1). Familiarity with the data was achieved through conducting interviews, engaging with the data during manual transcription, and undertaking repeated passes of the transcripts. The codebook was applied through reviewing all transcribed data and coding data extracts according to these four deductive codes. Data extracts that were relevant but could not be coded according to the existing domains were grouped and considered in subsequent analysis to ensure adequacy of the existing conceptualization of the surge capacity domains. Coded extracts were transferred to a data extraction table to facilitate the next stages of analysis.

**Table 1.** Description of Surge Capacity Domains as Deductive Codes

Code	Description
Staff	Personnel, including clinical and non-clinical
Stuff	Equipment and consumables, including clinical and non-clinical
Space	Physical space equipped with infrastructure
Systems	Collective and integrated processes, procedures, and regulations

Data extracts were then analyzed to allow new codes to emerge within the deductively applied codes, including the data extracts that could not be coded to a domain. Emerging codes were then considered for their commonality across domains and developed to identify the relevant underlying meaning. These underlying meanings were finally defined, and descriptions produced, thus allowing an understanding of their significance and relationship to the surge capacity domains to emerge.

### Ethical Considerations

Ethical approval was provided by the Human Research and Ethics Committee of Griffith University (Gold Coast, Queensland, Australia; Ref: 2023/281). Informed consent was required and participation was voluntary. There were no offers of reward or financial compensation for participation. Pseudonyms were used in analysis and reporting, with identifying statements are characteristics removed to preserve confidentiality.

## Results

Seventeen individuals participated in semi-structured interviews. Characteristics of the individuals are presented in Table 2.

**Table 2.** Summary of Participant Characteristics

Characteristic	Details
Profession	Doctor (Physician, Pediatrician), Doctor (Proceduralist), Manager (clinical), Manager (non-clinical), Nurse, Paramedic, Pharmacist
Region of Origin	Africa, Asia, Central America, Europe, Oceania, United States of America
Response Trigger	Armed Conflict, Earthquake, Famine, Flood, Infectious Disease Outbreak, Poverty, Tsunami, Typhoon
Response Location	Afghanistan, Angola, The Balkans, Cameroon, Central America, Colombia, Democratic Republic of Congo, Ethiopia, Gaza (historic, present as of 2024), Greek Islands, Haiti, Indonesia, Iraq, Jordan, Kenya, Lebanon, Liberia, Yemen, Mediterranean region, Mozambique, Myanmar, Nepal, North Korea, Pakistan, Palestine, Philippines, Somalia, Sudan and South Sudan, Thailand, Turkey, Uganda, Ukraine, Yemen

Staff, stuff, space, and systems were all represented by coded data extracts, demonstrating relevance of this conceptualization in the context of humanitarian response settings. Extracts that were relevant but could not be coded did not suggest insufficiency of the current four domains, but rather a lack of specificity regarding the relevant domain or domains when describing a response capability more collectively. Four cross-cutting themes, sponsorship, suitability, security, and supply - termed the “surge capacity dimensions” - were identified from the conducted latent content analysis, as outlined in Table 3.

**Table 3.** Description of Surge Capacity Dimensions as Emerging Themes

Dimensions	Description
Sponsorship	Political or legal: asset is accredited, endorsed, and is permitted within relevant legal frameworks/regulations
	Organizational: asset is within the capabilities of, and endorsed for use and managed by, the organization
	Financial: the asset is economically supported, funded
Suitability	Asset is fit for purpose and appropriate within the context
Security	Asset is secure or defendable against threats/harm/destruction
Supply	Asset is present or appropriately available on demand

### Sponsorship

For an asset or resource to render a positive impact in a humanitarian response, it must first have the required sponsorship. Emerging findings suggested three primary forms or levels of sponsorship: (1) political or level sponsorship; (2) organizational sponsorship; and (3) financial sponsorship. Participant experiences revealed that there is little future option or ability to operate effectively without all three forms of sponsorship, although potentially in varying degree depending on the asset.

Political and legal sponsorship requires that an asset or resource is accredited, endorsed, and permitted within the relevant legal frameworks and regulations. Participant experiences highlighted political sponsorship through identification of the consequences in instances where a lack of political approval became problematic or inhibiting:

*We may have someone who’s very qualified and particularly qualified in conflict medicine from these other countries and they aren’t able to get a visa past 90 days.* (P9)

*The exception being tricky medication – opiates … we actually ran out at one point and it is because of all of the importation regulations, nothing to do with the logistics chain and lack of resources. We simply were not allowed to import it into the country.* (P10)

Experiences also considered the effects of political influence and politically driven decisions on the ability to deliver effective and meaningful humanitarian assistance:

*[They] brought in three [groups] … who were not pre-registered … they didn’t know what they were doing. … You get political manipulation as well … They should not be giving you cover. It was political circumstances, it was a political decision rather than a medical decision.* (P12)

Organizational sponsorship requires that an asset or resource falls within the capabilities of an organization and has the required endorsements and management mechanisms to be operational and effective within the organizational context:

*The overwhelming work and expense and expertise in [the organization], it’s not medical – there are [tuberculosis] advisors and HIV advisors … it is supply and security, construction, water engineers, sanitation engineers…* (P6)

*Most of the major government field hospitals are coming in off the back of their own defense forces … if we needed something, it had to just come in on the back of a C130.* (P5)

*The ICRC [International Committee of the Red Cross; Geneva, Switzerland] has created very standard modules … for the equipment and consumables, and the WHO [World Health Organization; Geneva, Switzerland] has the International Emergency Health Kits and their standardized list of medications … It’s about having very standard equipment, consumables, drugs, and ensuring that each of the staff members sticks to the strategy and works towards the strategy …* (P9)

Finally, financial sponsorship requires that an asset or resource has economic support and funding to become and remain operational. This is highlighted by experiences in which financial support or control impacted operations or asset availability:

*… a donor will say “OK, you only have $500,000,” and that’s not going to cover all the team members. So, it’s about choosing which ones are right for that hospital, but we know we’re not going to actually achieve the outcomes that we are wanting …* (P9)

*Money always comes with strings attached and there’s always, particularly from your larger NGOs [non-government organizations] that are passing funding down to the smaller ones, they want you to do certain things with it.* (P8)

### Suitability

An adequately sponsored asset or resource must be fit for purpose and appropriate given the specific circumstances of the humanitarian response; the asset or resource therefore needs to be suitable.

Suitability related to staff considers qualifications, experiences, and even acceptability, including in terms of culture and ethnicity of relevant communities:

*… you may have an inexperienced surgeon, may have an experienced surgeon, but they may not have much experience in war wounds and how it is a different pathology from civilian trauma.* (P12)

*A big group of patients that was impossible to treat … [were] patients with complex wounds that would have required surgery. There was no OT [operating theatre], there was no surgeon; I’m an internal physician so I would not have dared to pick up any surgery … [no] surgical extraction of foreign bodies or similar.* (P3)

Stuff and space also highlight that suitability relates to the ability of the asset to bring about the required impact in the prevailing context. Suitability is therefore responsive not only to the context, but also to the goal or desired impact:

*This was meant to be an ICU [intensive care unit] – we had received ventilators and someone thought about that aspect … but we did not have the rest that you need for someone who is intubated… No suction immediately there to be suctioning patients when they are intubated. (P10)*

*… when we’re on the surgical mission, we were always there a day early … [to] walk through the hospital or the operating rooms and then put together what resources that they, that are going to be safe for us to use. … and it is that line in the sand - “well, that’s fine, but we’re not going to proceed unless we can effectively use this and this.”* (P7)

*When we are entering … they need an upgrade of the system, the cabling… In some places, there are fires because their system is old and there is no maintenance.* (P11)

Finally, reflections on systems further contribute recognition of the impact of system failures or shortcomings undermining system suitability:

*The doctor there had a lot of say in stuff as well, and he would get equally upset because he’d be ordering ahead, or flagging when things are running low, and still stuff wouldn’t come in. System failures …* (P1)

### Security

An adequately sponsored and suitable asset must then secure or defend against threats, harm, or destruction if it is to render a positive impact within the humanitarian response. Security here refers to protection from harm, including infectious diseases, and not in the way that the availability or supply of an asset is “secured” – this is the focus of the theme of *supply*.

Importantly, security need not be absolute, but rather sufficient given the specific circumstances and context of the humanitarian response. This balance between security and the humanitarian context can be seen in planning for and delivering humanitarian responses, influencing the humanitarian response:

*Every time we open a new project … someone from my team was the first going to the project, because if I don’t feel secure in what I’m doing there, or I don’t believe that the balance between insecurity and what we were doing was okay, then I would say “no, we cannot send expatriates there.”* (P14)

Security threats can impact the assets or resources already in place and render them incapable of bringing about their desired effect:

*… sometimes you are forced to hibernate yourself … the lack of access for people to reach health care facilities, the risk of attacks on health care … All of these things limit the overall operation.* (P16)

*You need to be careful with certain things because suddenly there is a warehouse that has been attacked and so we are missing those supplies, etc.* (P10)

Finally, challenges to security can, in some instances, be navigated through compensation, flexibility, or strategies that allow a humanitarian response to occur, but not without accepting additional burden compared to if security could be guaranteed:

*… that was an eight-hour drive … but with the gangs … you couldn’t drive on the road. … it would take a week for us to get four vans, four big lorries, because they’d have to leave at 02:00 in the morning when the gangs were asleep…* (P17)

### Supply

Finally, a sponsored, suitable, and adequately secure asset or resource must then be present or appropriately available on demand; the asset or resource must finally be supplied. Discussions of supply here considered that sponsorship, suitability, and security had been adequately addressed.

In relation to staff and stuff, supply refers to either “on-hand” or “within reach” availability to the degree to which the asset can render its desired impact:

*The ability to provide decent obstetric services … was contingent on the surgeon actually being there to do the caesarean. (P5)*

*Without a reliable supply, I would argue it’s medical tourism; you’re less than useless, just kind of wandering around the hospital for your own interests but not doing any good for anyone else.* (P6)

Supply in relation to space also considers existence and availability of the space required to render the required impact:

*Everything you can think of, from the most basic level, needs to be put in place to develop the health system or a hospital for that matter; a 100-bed hospital has to be put in …* (P6)

Finally, existence and availability of an operational and reliable system is also essential to sustaining an impactful response; however, this looks beyond a system for supply towards an existing complex of interconnected and functional elements required for operational success:

*We also know that more will come, because there is a big apparatus behind us and they will do everything, move heaven and earth, to make sure we get some new spinal needles and bandages.* (P10)

*… the bigger part of these projects, by a mile and far more than medicine, is logistics – it’s supply and supply chains. That’s far beyond the experience and expertise of any clinicians – you have logistics experts, human resources, security, and they are the bulk of the projects.* (P6)

### The 4S^2^ Model

**Figure 1.**
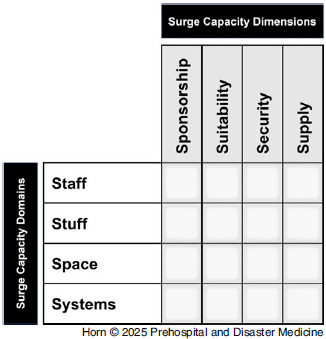
SHARE-HRS 4S^2^ Model: Surge Capacity Domains and Surge Capacity Dimensions.

The interactions between the original 4S surge capacity domains and the cross-cutting surge capacity dimensions (4S^2^ - cumulative 4S domains and the new dimensions) produce a four-by-four matrix, giving rise to the name of the SHARE-HRS 4S^2^ Model (Figure 1). In overlaying the domains and dimensions, every intersection between them was addressed by extracted data, with each presented extract first coded deductively according to the surge capacity domains before providing insight into the emerging dimension. The implications of the cross-cutting themes and intersections are that each of the dimensions must be addressed for the domain to render the required impact; therefore, as an example, staff able to render a positive contribution must have the required sponsorships, must be suitable, must be provided an appropriate degree of security, and finally must be supplied.

## Discussion

Deductively coded data demonstrate the applicability of existing surge capacity domains while also supporting the identification of four new themes, the surge capacity dimensions, namely: sponsorship, suitability, security, and supply. This research builds the evidence demonstrating the relevance of the “four Ss” conceptualization through purposeful exploration of scarce health resources in humanitarian response settings.^10^ Existing conceptualizations of surge capacity assert that each domain of staff, stuff, space, and systems must be adequately resourced to address a surge in care beyond an original operational baseline.^1–3^ To date, resourcing has been almost exclusively framed around the “availability” of “adequate” resources within each domain; for example, when operationalized, surge capacity supported by the staff domain imposes a requirement for the availability of adequate numbers of skilled staff.^4–8^ As noted, humanitarian response settings and complex humanitarian emergencies are often characterized by profound resource limitations, complex political influences, and conflict or other uses of violence, including against humanitarian workers.^9,18^ The results of this analysis support the translation of this dynamic between the domains, in that critical shortages arising from one domain limits overall capacity to humanitarian response settings; however, this exploration of humanitarian response contexts highlights a greater complexity than the presence or absence of assets within each domain.

The dimensions of sponsorship, suitability, security, and supply represent a novel advancement in existing surge capacity constructs in terms of health resources and humanitarian health response capability. The impacts of funding (financial sponsorship), resource availability, and reliable logistics supply chains in relation to procurement and distribution are well-recognized as impacting supply in humanitarian logistics research.^19,20^ However, exploration of surge capacity constructs highlights that these factors can support or prohibit specific humanitarian health responses beyond the maintenance of supply chains, which can be viewed as assets within the systems domain. Additionally, humanitarian funding is complex and impacted by a multitude of factors, notably including both political and organizational factors, which disrupt funding distribution based solely on need,^21^ the impacts of which are also recognized in humanitarian logistics research.^19^ This research therefore builds upon existing humanitarian logistics research by recognizing all four domains while simultaneously building upon surge capacity conceptualizations by more specifically defining the scope of “supply” and recognizing supply of assets alone is insufficient in humanitarian response contexts.

The potential relevance of security in surge capacity for humanitarian health responses has been previously suggested.^10^ Attacks on humanitarian workers are of growing concern, with perceived ambivalence to the protections afforded humanitarians by parties to conflict in recent and on-going events.^18,22,23^ Security from violence is additionally recognized in humanitarian logistics literature, including in terms of asset protection from hazards and threats other than that of violence.^19,20,24^ This exploration of lived experiences provides important context to the role and conceptual connection of security to a humanitarian health response capability, thus adding support to the proposed relevance of security in surge capacity conceptualizations.

### The 4S^2^ Model

**Figure 2.**
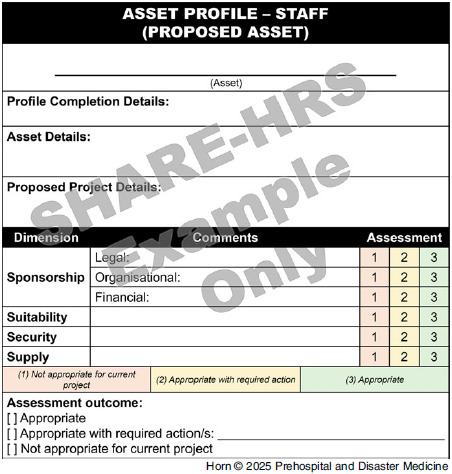
Example Recommendation for SHARE-HRS 4S^2^ Model Operationalization.

Establishing congruence between the derived surge capacity dimensions and existing literature further builds the credibility of these findings. This research, however, contributes a potential new tool for use in humanitarian response planning and evaluation by: consolidating existing surge capacity domains from other contexts that differ significantly from humanitarian response settings, demonstrating their relevance in these contexts; identifying and formalizing themes as they emerge from humanitarian response settings, adding a greater degree of detail than in previous conceptualizations; and unifying these concepts in a model directly informed by context-specific evidence, and consideration given to its potential implementation. As the 4S^2^ model emerged, several recommended applications also emerged, as summarized in Table 4. Additionally, Figure 2 contains an example recommendation for how the SHARE-HRS 4S^2^ model may be operationalized in a profile form to support the proposed applications in Table 4.

**Table 4.** Potential Applications of the SHARE-HRS 4S^2^ Model

Phase	Proposed Application	Example (Staff Domain)
Needs Assessment	Use of the model to identify, organize, and articulate critical assets and their characteristics (dimensions) required to address assessed needs.	Needs assessments reveal unmet surgical need faced by a deployed unit and thus the model dimensions may be used to describe a need for a general surgeon, with the specific licensing and permissions to work in-country, and having availability to fill the specific gap in capability. This information can be produced as a reference profile for the specific asset and can be used in informing strategic planning.
Strategic Planning	Use of the model to identify appropriate potential assets and support matching them to needs with structured justification. Profiles (reflecting the 4S^2^ domains) completed for a range of potential responders can be compared to the needs assessment profile, supporting consistent and transparent suitability assessment and approval for deployment.	Several potential surgeons may be compared to the needs assessment to identify the surgeon with characteristics most closely matched, and the overall appropriateness of a proposed asset can be clearly articulated to stakeholders (eg, management, economic funders, regulators).
Implementation and Monitoring	Cyclical use of the model to update needs assessments and strategic planning while iteratively evaluating the appropriateness of assets available and in use. Iterations of asset profiles and reiterative assessments of on-going suitability provide transparent, structured, and timely data for reporting.	Repeated comparison between the asset profile of the deployed surgeon and evolving needs assessments supports clear justification for either continued suitability or the need for a change in assets (such as an emerging need for specialist orthopedic surgical capability would reduce the suitability of the general surgeon in the deployed capability and may prompt a change in the deployed asset).
Review and Evaluation	After-action use of the model to evaluate the appropriateness of assets utilized in a response, using the dimensions to articulate where barriers to appropriateness or effectiveness arose, or to provide structured data to support the success of the deployment.	An evaluation of how assets addressed evolving needs, how security threats challenged the appropriateness of deployed capability for practitioners, and to provide standardized data for research.

The 4S^2^ model therefore provides a proposed common nomenclature and conceptualization of surge capacity in humanitarian response settings. Additionally, the 4S^2^ model has practical implications with interactions being expandable to include all relevant potential barriers and considerations, both in planning (anticipatory) and evaluation (retrospective). Given its grounding in existing surge capacity conceptualizations, further consideration should be given to the transferability of the 4S^2^ model back to non-humanitarian response contexts.

## Limitations

Sampling driven by maximum variation means that these findings emerged as commonalities across a broad spread of participants and settings. Additionally, despite applying deductive methods, this research remained fundamentally exploratory. Therefore, these findings, including the produced SHARE-HRS 4S^2^ model, emerge as evidence-based and valid findings but were not tested through confirmatory methods within each of the included contexts, nor was in-depth context-specific validation sought.

The overall research project is underpinned by pragmatist perspectives and abductive reasoning. This orientates the research to the production of impactful and usable outputs while accepting that future revisions may be required in response to future evidence. Therefore, future research into context-specific applications represents an important next step for the SHARE-HRS 4S^2^ model as a model for both research and practice.

## Conclusions

Both surge capacity domains and newly emerged surge capacity dimensions are required to appropriately represent and understand surge capacity conceptualizations in humanitarian response settings. Overlaid, these domains and dimensions form a four-by-four matrix and thus the SHARE-HRS 4S^2^ model emerged as a conceptualization of surge capacity specific to scarce health resources in humanitarian response settings. This novel model provides an important advancement in understanding and managing scarce health resource allocation in these challenging settings, as well as offering a potential new framework to support future practice and research in this area.

## Supporting information

Horn et al. supplementary material Horn et al. supplementary material

## Supplementary Materials

To view supplementary material for this article, please visit https://doi.org/10.1017/S1049023X25101210
