# Supplementary material for: The SHARE-HRS 4S2 Model of Surge Capacity in Humanitarian Health Care Response Settings: A Revised Model Informed by Lived Experiences
Source: Prehosp Disaster Med. 2025 Jun 26;40(3):162–8. doi: 10.1017/S1049023X25101210 (PMC12237698; doi:10.1017/S1049023X25101210)
Supplement: Horn et al. supplementary material [file S1049023X25101210sup001.docx]

**Real-World Experiences of Health Resource Allocation in Humanitarian Responses – Part II**

**Semi-Structured Interview Guide**

Date: __/__/__ Time: __:__ hrs AEST Interviewee: _____ Audio: ____

**Notes**

***Participant Introduction (Ice-breaker):*** Could you please start by telling me about yourself?

- Prompts may include the following points: (1) clinical/professional role; (2) previous academic/clinical training; (3) previous clinical experience; (4) overall summary of humanitarian response experience/s.

***Nature of Relevant Experience:*** Thinking about 1 or 2 of your major experiences in humanitarian responses, can you tell me your story from when you found out about the event or your potential involvement in the response?

- Prompt: Please tell me *more* about the nature of the responses to humanitarian crisis/crises you have been a part of. *(Aim to elicit information around the event, including type/nature/location, and individual role including duration)*
- Prompt: Please tell me about how you came to participate in this/these responses. *(Aim to elicit information around the nature of the response – NGO, government, military etc – specific organisation names to be excluded for confidentiality)*

**The following prompts relate to specific lines of enquiry if required to direct focused exploration.**

***Resource Allocation:*** Please tell me about a time when you had a role in allocating a scarce resource during this (*or* one of these) response/s.

- **Prompt:** Please tell me more about your specific role in the decision-making processes used to allocate scarce resources *(Were you responsible for allocation decision-making; if not the participant, then who was making allocation decisions; how did the participant interact with the decision-making process)*.
- **Prompt:** Please tell me about other decision / factors that impacted whether resources were available or how they were allocated.

***Denial of Care:*** Can you think of any specific examples where resource scarcity meant individuals were denied resources? Please tell me about this.

- **Prompt:** Please tell me about the specific resource/s that came to require resources to be denied to individuals.
- **Prompt:** Please tell me about the ways in which it was decided who would receive, or be denied, resources/care.
- **Prompt:** Were there individuals or groups that were prioritised for care? Were there individuals or groups that were routinely denied care based on characteristics? What are these?
- **Prompts:**
  - Can you recall any ways in which resource allocation decisions were guided?
  - Were guidelines or structures (formal or informal) provided?
  - Please tell me about how you personally interacted / utilised / experienced / perceived these guidelines/structures.

***Closing Question:*** We are coming to the end of the interview. Is there anything else you would to mention that we haven’t discussed, or a take-away message that you think is particularly important?

**Notes on Interview Guide/Structure/Technique:**

__________________________________________________________________________________________________________________________________________________________________________________________________________________________________________________________________________________________________________________
